# Routine delivery of artemisinin-based combination treatment at fixed health facilities reduces malaria prevalence in Tanzania: an observational study

**DOI:** 10.1186/1475-2875-11-140

**Published:** 2012-04-30

**Authors:** Rashid A Khatib, Jacek Skarbinski, Joseph D Njau, Catherine A Goodman, Berty F Elling, Elizeus Kahigwa, Jacquelin M Roberts, John R MacArthur, Julie R Gutman, Abdunoor M Kabanywanyi, Ernest E Smith, Masha F Somi, Thomas Lyimo, Alex Mwita, Blaise Genton, Marcel Tanner, Anne Mills, Hassan Mshinda, Peter B Bloland, Salim M Abdulla, S Patrick Kachur

**Affiliations:** 1 Ifakara Health Institutce, Dar-es-Salaam, Tanzania; 2 Swiss Tropical and Public Health Institute, Basel, Switzerland; 3 Malaria Branch, Centers for Disease Control and Prevention, Atlanta, USA; 4 Rollins School of Public Health, Emory University, Atlanta, USA; 5 Kenya Medical Research Institute/Wellcome Trust Research Programme, Nairobi, Kenya; 6 London School of Hygiene and Tropical Medicine, London, UK; 7 Karolinska Institutet, Stockholm, Sweden; 8 Swiss Development Cooperation, Dar-es-Salaam, Tanzania; 9 Australian Centre for Economic Research on Health, Australian National University, Canberra, Australia; 10 Ministry of Health and Social Welfare, Dar-es-Salaam, Tanzania; 11 Tanzania Commission on Science and Technology, Dar-es-Salaam, Tanzania

**Keywords:** Malaria, Artemisinin-based combination therapy, Transmission reduction, Malaria

## Abstract

**Background:**

Artemisinin-based combination therapy (ACT) has been promoted as a means to reduce malaria transmission due to their ability to kill both asexual blood stages of malaria parasites, which sustain infections over long periods and the immature derived sexual stages responsible for infecting mosquitoes and onward transmission. Early studies reported a temporal association between ACT introduction and reduced malaria transmission in a number of ecological settings. However, these reports have come from areas with low to moderate malaria transmission, been confounded by the presence of other interventions or environmental changes that may have reduced malaria transmission, and have not included a comparison group without ACT. This report presents results from the first large-scale observational study to assess the impact of case management with ACT on population-level measures of malaria endemicity in an area with intense transmission where the benefits of effective infection clearance might be compromised by frequent and repeated re-infection.

**Methods:**

A pre-post observational study with a non-randomized comparison group was conducted at two sites in Tanzania. Both sites used sulphadoxine-pyrimethamine (SP) monotherapy as a first-line anti-malarial from mid-2001 through 2002. In 2003, the ACT, artesunate (AS) co-administered with SP (AS + SP), was introduced in all fixed health facilities in the intervention site, including both public and registered non-governmental facilities. Population-level prevalence of *Plasmodium falciparum* asexual parasitaemia and gametocytaemia were assessed using light microscopy from samples collected during representative household surveys in 2001, 2002, 2004, 2005 and 2006.

**Findings:**

Among 37,309 observations included in the analysis, annual asexual parasitaemia prevalence in persons of all ages ranged from 11% to 28% and gametocytaemia prevalence ranged from <1% to 2% between the two sites and across the five survey years. A multivariable logistic regression model was fitted to adjust for age, socioeconomic status, bed net use and rainfall. In the presence of consistently high coverage and efficacy of SP monotherapy and AS + SP in the comparison and intervention areas, the introduction of ACT in the intervention site was associated with a modest reduction in the adjusted asexual parasitaemia prevalence of 5 percentage-points or 23% (p < 0.0001) relative to the comparison site. Gametocytaemia prevalence did not differ significantly (p = 0.30).

**Interpretation:**

The introduction of ACT at fixed health facilities only modestly reduced asexual parasitaemia prevalence. ACT is effective for treatment of uncomplicated malaria and should have substantial public health impact on morbidity and mortality, but is unlikely to reduce malaria transmission substantially in much of sub-Saharan Africa where individuals are rapidly re-infected.

## Background

Malaria continues to be a major cause of morbidity and mortality in sub-Saharan Africa. Vector control interventions and case management continue to be the cornerstones of malaria control efforts [1,2]. In recent years, artemisinin-based combination therapy (ACT) has been introduced for malaria case management in nearly all countries in sub-Saharan Africa, based mostly on its clinical efficacy at the individual level. However, part of the promise of ACT-based case management has been the potential to reduce malaria transmission through the gametocytocidal effects of ACT [3-8] as well as the post-treatment prophylactic effect of the partner drug [9]. Although several reports have suggested the impact of ACT on population-level malaria burden, most have used a pre-post design without a comparison group, have failed to account for the influences of concomitant interventions, such as indoor residual spraying and ecological changes that may have also reduced transmission, or have been limited to areas of low malaria transmission [10-15]. Few studies have been designed to quantify the independent impact of ACT-based case management policy on malaria prevalence in areas of high malaria transmission by incorporating contemporaneous observational outcomes in areas without the benefit of ACT.

Malaria transmission is dependent on mosquito vector dynamics, the proportion of humans with peripheral gametocytaemia, and the infectiousness of circulating gametocytes to mosquitoes. The relationship between malaria endemicity, the prevalence of the disease in the population, and transmission has been defined for moderate to high transmission settings [16]. Asexual parasitaemia prevalence continues to be an accepted measure of malaria endemicity in high transmission settings and is a measureable human correlate of malaria transmission intensity in the population [17]. Unfortunately, more precise human correlates of transmission intensity, such as incidence, are difficult to measure in high transmission settings and in studies of case management interventions. If the introduction of ACT with its gametocytocidal properties has an impact on malaria transmission, it should be possible to detect a reduction in asexual parasitaemia prevalence following its widespread uptake.

In 2003, the Interdisciplinary Monitoring Project for Anti-Malarial Combination Therapy in Tanzania (IMPACT-Tz) assisted the Ministry of Health to implement artesunate (AS) plus sulphadoxine-pyrimethamine (SP) combination treatment at all 56 health facilities in Rufiji District as the first-line treatment for uncomplicated malaria, while the official first-line anti-malarial was still SP monotherapy in the rest of Tanzania, having been rolled out nationwide in August 2001. The primary objective of IMPACT-Tz was to evaluate the feasibility of delivering ACT through routine health systems as well as the potential of AS + SP combination treatment to prevent the spread of SP drug resistance and reduce malaria transmission [18,19]. In this paper, the impact of introducing AS + SP in public and registered non-governmental organization health facilities on population asexual parasitaemia and gametocytaemia prevalence is explored. This is the first study to assess the population-level impact of introducing ACT in an observational setting with a contemporaneous comparison group.

## Methods

### Study site

The study was conducted in two Demographic and Health Surveillance System (DHSS) sites, Kilombero-Ulanga DHSS and Rufiji DHSS, in southern Tanzania. The Kilombero-Ulanga DHSS had 72,000 population [20] while the Rufiji DHSS had 85,000 population [21]. Both sites are situated in the Greater Rufiji River Basin, experience perennial malaria transmission, and are primarily rural with the majority of the population relying on subsistence farming or fishing. Both sites have been used for other malaria studies; most importantly, the Kilombero-Ulanga site was used for a study on the social marketing of ITNs [22] and ITN use was substantially higher in this site compared to the Rufiji site at the start of this evaluation in 2001. Social marketing of ITNs, along with subsidized distribution through a voucher system in antenatal clinics and supplementary free mass distribution campaigns, increased coverage of ITNs in both study sites by 2006 [23-26]. Both sites were used for the Integrated Management of Childhood Illness Multi-Country Evaluation with Rufiji serving as an intervention area and Kilombero-Ulanga as the comparison area [27]. Lastly, the Kilombero-Ulanga DHSS was used for the ACCESS Programme from 2004 to 2008, a quality improvement project aimed at enhancing access to prompt and effective malaria treatment and care through social marketing for improved care-seeking and quality of care at health facilities [28-31].

The health system in both sites was comprised of a network of hospitals, health centers and dispensaries operated by the government of Tanzania and non-governmental organizations. Most persons obtained anti-malarials from health facilities, drug shops or general shops. Most anti-malarial prescribing was based on clinical diagnosis alone and few patients received anti-malarials based on laboratory-confirmed parasitaemia.

### Study design

A pre-post observational study with a non-randomized comparison group was undertaken over six years. Both sites used SP monotherapy as a first-line anti-malarial from 2001–2002. According to recommendations of the National Malaria Control Programme [32], SP monotherapy was dispensed in seven age- or weight-specific dosing bands to achieve a target total sulphadoxine dose of 25 mg/kg. It was further recommended that SP be given as a single stat treatment dose under direct observation in the health facility. In March 2003, the Council Health Management Team implemented AS + SP combination therapy as the first-line anti-malarial delivered through all the fixed health facilities in Rufiji District alone. As had been done in ACT efficacy studies, AS + SP was dispensed in four age- and weight-specific dosing bands to achieve a total target dose of 25 mg/kg sulphadoxine stat and 12 mg/kg artesunate divided in 3 daily doses [33]. The complete dose of SP and the first daily dose of AS were delivered under direct observation in the health facility, and the patient was sent home with remaining doses of AS in preprinted dosing envelopes [34]. SP monotherapy continued to be the first-line anti-malarial in Kilombero-Ulanga, the comparison site, as well as in the rest of the country. Trends in prevalence of asexual parasitaemia and gametocytaemia were observed across both sites before and after the introduction of AS + SP in Rufiji.

### Implementation of AS + SP

As part of IMPACT-Tz, World Health Organization (WHO) prequalified AS tablets (Arsumax® 50 mg, Sanofi, Gentilly, France) were supplied to the Rufiji site and relied on existing systems for the delivery of SP. At the time, no co-packaged or co-formulated ACT product was available and SP + AS was co-administered at the point of care. In addition, all health workers providing clinical services in Rufiji District were equipped with training and job-aids to support the ACT use, first in 2003 and again in 2005. AS supply was closely monitored to minimize stock-outs. Stock-outs were rare until 2006 when delays in introducing artemether-lumefantrine as first-line therapy, originally planned in 2004, led to shortages of both AS (in Rufiji) and SP (throughout all of mainland Tanzania).

### Household survey data collection procedures

Cross-sectional household surveys were completed in both sites in 2001, 2002, 2004, 2005, and 2006. Households were randomly selected from the two DHSS sites covering a combined area of 29,000 km^2^ and including communities as far as 300 km apart. Independent samples from the same 56 census enumerated villages (31 in Rufiji and 25 in Kilombero-Ulanga) were selected for each survey year. All surveys were conducted between June and September, which coincides with the end of the long rainy season when malaria is reportedly at its peak. A standardized questionnaire to measure internationally recognized indicators [35] was administered to the head of the household and individual household members. Study participants were individually asked for written informed consent. For children less than 12 years old, consent was obtained from the parent or guardian. A finger prick blood sample was taken from every member of the household available on the day of the visit and a blood slide collected. Blood slides were sent to an Ifakara Health Institute reference laboratory where they were stained with Giemsa and read by trained microscopists using standard procedures for preparation, interpretation and reporting, as described earlier [36]. Both *P. falciparum* and non-falciparum asexual parasites and gametocytes were identified, but over 98% of malaria infections in these areas were due to *P. falciparum* and prevalence of nonfalciparum infection is not reported. Asexual parasites and gametocytes were quantified by counting number of parasites per 500 white blood cells. Parasite density was estimated by assuming a count of 8,000 white blood cells per microlitre. Five percent of slides read by each microscopist were read again by a senior laboratory technician for quality control; discordant readings were consistently less than 14%.

### *In vivo* study data collection and analysis

Standard *in vivo* efficacy studies based on 1996 and 2003 WHO protocols [37,38] were also completed in both Rufiji and Kilombero-Ulanga DHSSs in 2001, 2002, 2004, and 2006. Children <5 years old with documented fever (axillary temperature >37.5°C) in the absence of another obvious cause of fever, and mono-infection with *P. falciparum* of between 2,000 and 250,000 asexual parasites/mm^3^ as determined by microscopic examination of a peripheral blood smear were enrolled and informed consent obtained. Children with signs of severe malaria, or reported history of allergy to anti-malarial or sulpha drugs, were excluded. Patients were randomly allocated to receive standard doses of either SP or AS + SP [37]. Patients were followed up on days 1, 2, 3, 7 and 14 in all years. To standardize analysis across all years and sites, all *in vivo* data were analysed using modified 2003 WHO definitions of clinical and parasitological failure up to day 14 [37], as well as the 1996 WHO definition of adequate clinical response at day 14 [38], which was the prevailing recommendation at the onset of the study. Polymerase chain reaction correction was not performed until 2004, and those results are not presented here.

### Ethical approval

Ethical approval for the studies was obtained from the institutional review boards of Ifakara Health Research and Development Centre (IHRDC, now Ifakara Health Institute), the United States Centers for Disease Control and Prevention (CDC), the London School of Hygiene and Tropical Medicine, and the National Tanzanian Medical Research Co-ordinating Committee of the National Institute for Medical Research.

### Data analysis

Data were double entered using Microsoft FoxPro software (Redmond, Washington, United States) and analysed using SAS version 9.2 (SAS Institute, Cary, North Carolina, United States). Descriptive analyses were done using the survey analysis tools, which use the Taylor expansion method to account for household-level clustering. Comparisons of proportions were done using the Wald chi square. Univariate and multivariate logistic regression modelling were performed using the surveylogistic procedure. Statistical significance was defined as a p-value ≤0.05. An index of socio-economic status was generated for each survey year separately using principal components analysis for household characteristics and asset ownership as described elsewhere [39]. Monthly rainfall data were collected from weather stations adjacent to the respective DHSS sites. A conservative estimate of the annual population drug pressure defined as the number of anti-malarial treatments per 100 persons per year was calculated by multiplying the proportion of persons who reported receiving an anti-malarial during the two week recall period of the survey by 26, the number of two-week periods per year. This estimate assumes constant anti-malarial use throughout the year.

Multivariate logistic regression was used to assess the effect of implementation of AS + SP on asexual parasitaemia and gametocytaemia. Using a model with terms for study group (intervention versus comparison), survey year, and intervention (implementation of AS + SP in Rufiji in 2004–2006), changes in asexual parasitaemia and gametocytaemia prevalence were compared between the pre-intervention and post-intervention surveys, as well as changes in their relative difference (i.e. change in the intervention group between pre- and post-surveys versus change in the comparison group). The model was adjusted for potential confounding by including age group, untreated bed net and ITN use, socioeconomic status, and rainfall in the six months prior to interview.

### Role of funding source

The sponsor of the study had no role in study design, data collection, data analysis, data interpretation, or writing of the report. The corresponding author had full access to all of the data in the study and had final responsibility for the decision to submit for publication.

## Results

The surveys included 38,872 persons; but analysis is limited to the 37,309 (96%) persons for whom complete data were available. The age and socioeconomic status distributions were similar across sites and years (Table 1). However, use of untreated bed nets and ITNs was consistently higher in Kilombero-Ulanga than Rufiji. Mean rainfall varied from year to year and was often higher in Kilombero-Ulanga compared to Rufiji.

**Table 1:** Characteristics of study populations in Rufiji and Kilombero-Ulanga (KU) Demographic and Health Surveillance System sites, Tanzania, 2001, 2002, 2004, 2005, 2006 (N = 37,309)

	**2001**		**2002**		**2004**		**2005**		**2006**	
	**Rufiji (N = 1,521)n (%)**	**KU (N = 1,543)n (%)**	**Rufiji (N = 2,956) n (%)**	**KU (N = 3,885)n (%)**	**Rufiji (N = 3,715) n (%)**	**KU (N = 4,044)n (%)**	**Rufiji (N = 5,399) n (%)**	**KU (N = 4,902)n (%)**	**Rufiji (N = 4,190) n (%)**	**KU (N = 5,223)n (%)**
Median age in years (interquartile range)	16 (6–39)	19 (7–35)	15 (6–36)	17 (6–34)	14 (5–35)	15 (5–35)	14 (5–35)	15 (5–33)	14 (5–34)	15 (6–34)
**Age groups**										
<1 year old	38 (2%)	44 (3%)	81 (3%)	95 (2%)	100 (3%)	156 (4%)	128 (2%)	207 (4%)	157 (4%)	163 (3%)
1–4 years old	229 (15%)	209 (14%)	453 (15%)	628 (16%)	639 (17%)	652 (16%)	957 (18%)	820 (17%)	675 (16%)	781 (15%)
5–15 years old	466 (31%)	432 (28%)	929 (31%)	1115 (29%)	1180 (32%)	1201 (30%)	1717 (32%)	1473 (30%)	1369 (33%)	1629 (31%)
>15 years old	788 (52%)	858 (56%)	1493 (51%)	2035 (53%)	1796 (48%)	2035 (50%)	2597 (48%)	2402 (49%)	1989 (47%)	2618 (50%)
**Bed net use previous night**										
Used untreated net	235 (15%)	911 (59%)	437 (15%)	2312 (60%)	416 (11%)	2063 (51%)	911 (17%)	2302 (47%)	1430 (34%)	2640 (51%)
Uses insecticide treated net	42 (3%)	145 (9%)	70 (2%)	395 (10%)	370 (10%)	1045 (26%)	1197 (22%)	1688 (34%)	1300 (31%)	1864 (36%)
**Socioeconomic status by asset index**										
Poorest	287 (19%)	394 (26%)	410 (17%)	629 (17%)	625 (17%)	787 (19%)	853 (16%)	844 (17%)	593 (14%)	942 (18%)
Less poor	163 (11%)	224 (15%)	337 (14%)	715 (19%)	823 (22%)	624 (15%)	1074 (20%)	1004 (20%)	816 (19%)	998 (19%)
Middle	281 (18%)	332 (22%)	496 (20%)	804 (21%)	805 (22%)	766 (19%)	1159 (21%)	958 (20%)	938 (22%)	1048 (20%)
More rich	416 (27%)	249 (16%)	589 (24%)	830 (22%)	726 (20%)	984 (24%)	1224 (23%)	977 (20%)	903 (22%)	1125 (22%)
Least poor	374 (25%)	344 (22%)	613 (25%)	799 (21%)	736 (20%)	883 (22%)	1089 (20%)	1119 (23%)	940 (22%)	1079 (21%)
**Mean rainfall in previous 6 months from date of interview (in cm)**	61.3	44.2	96.4	198.4	74.5	149.0	67.6	146.1	93.3	136.1
**Malaria burden**										
Asexual parasitaemia	400 (26%)	274 (18%)	828 (28%)	851 (22%)	696 (19%)	1013 (25%)	999 (19%)	559 (11%)	633 (15%)	698 (13%)
Geometric mean asexual parasite density (95% CL)	886 (746,1052)	1008 (830,1222)	821 (736,916)	1032 (923,1152)	1104 (969,1259)	992 (895,1100)	1122 (1008,1248)	727 (637,831)	2144 (1837,2504)	540 (482,605)
Gametocytaemia	30 (2%)	23 (1%)	44 (1%)	69 (2%)	23 (1%)	73 (2%)	47 (1%)	14 (<1%)	14 (<1%)	25 (<1%)
Geometric mean gametocyte density (95% CL)	66 (50,86)	113 (69,186)	104 (60,183)	97 (76,123)	76 (46,125)	91 (74,113)	67 (46,99)	67 (38,121)	224 (95,528)	128 (48,343)
Gametocytemic patients of parasitemic pts	30/400 (7.5%)	23/274 (8.4%)	44/828 (5.3%)	69/851 (8.1%)	23/696 (3.3%)	73/1013 (7.2%)	47/999 (4.7%)	14/559 (2.5%)	14/633 (2.2%)	25/698 (3.6%)
**Fever in prior two weeks**	301 (20%)	227 (15%)	339 (11%)	571 (15%)	377 (10%)	421 (10%)	756 (14%)	484 (10%)	457 (11%)	462 (9%)
**Health seeking and anti-malarial use for febrile illness in prior two weeks**										
Use of health facility	89 (6%)	57 (4%)	115 (4%)	187 (5%)	160 (4%)	107 (3%)	299 (6%)	163 (3%)	130 (3%)	193 (4%)
Use of any anti-malarial	69 (5%)	78 (5%)	98 (3%)	242 (6%)	152 (4%)	212 (5%)	270 (5%)	267 (5%)	117 (3%)	287 (6%)
Use of sulphadoxine-pyrimethamine only	25 (2%)	6 (<1%)	65 (2%)	144 (4%)	41 (1%)	104 (3%)	36 (1%)	120 (2%)	42 (1%)	136 (3%)
Use of artesunate-sulphadoxine-pyrimethamine	0 (0%)	0 (0%)	0 (0%)	0 (0%)	83 (2%)	0 (0%)	180 (3%)	0 (0%)	32 (1%)	0 (0%)
Use of artesunate monotherapy	0 (0%)	0 (0%)	0 (0%)	0 (0%)	2 (<1%)	0 (0%)	5 (<1%)	0 (0%)	10 (<1%)	0 (0%)
Use of other anti-malarial*	44 (3%)	72 (5%)	33 (1%)	98 (3%)	27 (1%)	108 (3%)	52 (1%)	147 (3%)	33 (1%)	151 (3%)
**Estimated number of anti-malarial treatments per 100 persons per year**^**†**^										
Any anti-malarial	118	131	86	162	106	136	130	142	73	143
Sulphadoxine-pyrimethamine only	43	10	57	96	29	67	17	64	26	68
Artesunate-sulphadoxine-pyrimethamine	0	0	0	0	58	0	87	0	20	0
Other anti-malarial*	75	121	29	66	19	69	25	80	20	75

*Primarily quinine, amodiaquine and very rarely chloroquine.

^**†**^ An estimate of the annual population drug pressure defined as the number of anti-malarial treatments per 100 persons per year was calculated by multiplying the proportion of persons receiving an anti-malarial during the two week recall period of the survey by 26 (the number of 2 week periods per year).

The prevalence of fever varied significantly by year and site with a range of 9 to 20% reporting fever in the prior two weeks. About 3–5% of the entire population was treated with any anti-malarial for an episode of fever in the prior two weeks and the estimated annual drug pressure varied between 73 and 162 anti-malarial treatments per 100 persons per year. In Rufiji in 2004–2006, the annual population drug pressure using AS + SP varied from 20 to 87 AS + SP treatments per 100 persons per year. AS + SP delivered through public and non-governmental organization health facilities accounted for 27% to 67% of all anti-malarial treatments reported by persons with a febrile illness in the prior two weeks in Rufiji from 2004–2006. SP and AS + SP efficacy was assessed using modified *in vivo* studies. Both anti-malarial regimens were efficacious in 2001 with an adequate clinical and parasitological response at day 14 (ACPR) of 91% for SP and 97–100% for AS + SP. SP monotherapy was noted to have declining efficacy with an ACPR of 65–86% in 2004 and 2006 and had moderate levels of adequate clinical response (77%–92%) as it had been defined in 1996 (Table 2). AS + SP continued to be highly efficacious with an ACPR of 89–100% in 2004 and 2006.

**Table 2:** ***In vivo *efficacy of sulphadoxine-pyrimethamine (SP) and artesunate-sulphadoxine-pyrimethamine (AS + SP) for the treatment of uncomplicated malaria in children <5 years old in Rufiji and Kilombero-Ulanga (KU) Demographic and Health Surveillance System sites, Tanzania, 2001, 2002, 2004, 2006 **

	**2001 **		**2002 **		**2004 **		**2006 **	
	**Rufiji**	**KU**	**Rufiji**	**KU**	**Rufiji**	**KU**	**Rufiji**	**KU**
**Sulphadoxine-pyrimethamine**	**(N = 74)**	**(N = 66)**	**(N = 87)**	**(N = 77)**	**(N = 66)**	**(N = 65)**	**(N = 57)**	**(N = 52)**
**Early treatment failure (ETF)**^**1**^	0%	5%	6%	0%	11%	5%	4%	13%
**Late treatment failure**								
**Late clinical failure (LCF)**^**2**^	1%	0%	0%	0%	8%	3%	19%	10%
**Late parasitological failure (LPF)**^**3**^	8%	5%	18%	8%	5%	6%	7%	12%
**Adequate Clinical Response (ACR)**	99%	95%	94%	100%	82%	92%	77%	77%
**Adequate Clinical and Parasitological Response (ACPR)**^**4**^**at Day 14**	91%	91%	76%	92%	77%	86%	70%	65%
**Artesunate-sulphadoxine-pyrimethamine**	**(N = 67)**	**(N = 59)**	**(N = 86)**	**(N = 78)**	**(N = 72)**	**(N = 66)**	**(N = 58)**	**(N = 57)**
**Early treatment failure (ETF)**^**1**^	0%	0%	5%	0%	0%	0%	0%	0%
**Late treatment failure**								
**Late clinical failure (LCF)**^**2**^	0%	0%	0%	0%	0%	0%	5%	7%
**Late parasitological failure (LPF)**^**3**^	0%	3%	9%	4%	3%	0%	2%	4%
**Adequate Clinical Response (ACR)**	100%	100%	95%	100%	100%	100%	95%	93%
**Adequate Clinical and Parasitological Response (ACPR)**^**4**^**at Day 14**	100%	97%	86%	96%	97%	100%	93%	89%

^1^ Early treatment failure defined as: development of danger signs or severe malaria on day 1, day 2 or day 3 in the presence of parasitaemia; or parasitaemia on day 2 higher than day 0 count irrespective of axillary temperature; or parasitaemia on day 3 with axillary temperature ≥37.5°C; or parasitaemia on day 3 ≥25% of count on day 0.

^2^ Late clinical failure defined as development of danger signs or severe malaria on any day from day 4 to day 28 in the presence of parasitaemia, without previously meeting any of the criteria of Early Treatment Failure; or presence of parasitaemia and axillary temperature ≥37.5°C on any day from day 4 to day 14, without previously meeting any of the criteria of Early Treatment Failure.

^3^ Late parasitological failure defined as presence of parasitaemia on any day from day 7 to day 14 and axillary temperature <37.5°C, without previously meeting any of the criteria of Early Treatment Failure or Late Clinical Failure.

^4^ Adequate clinical and parasitological response at day 14 defined absence of parasitaemia on day 14 irrespective of axillary temperature without previously meeting any of the criteria of Early Treatment Failure or Late Clinical Failure or Late Parasitological Failure.

Note: In 2001, 4 patients were lost to follow-up from Rufiji with 2 in the SP group and 2 in the AS + SP group, while 7 patients were lost in KU with 4 in the SP group and 3 in the AS + SP group. In 2004, 12 patients were lost in Rufiji with 9 in the SP group and 3 in the AS + SP group; 5 were lost in KU with 3 in the SP group and 2 in the AS + SP group. In 2006, 5 patients were lost to follow-up in Rufiji with 2 in the SP group and 3 in the AS + SP group; 14 were lost in KU with 10 in the SP group and 4 in the AS + SP group.

**Figure 1:**
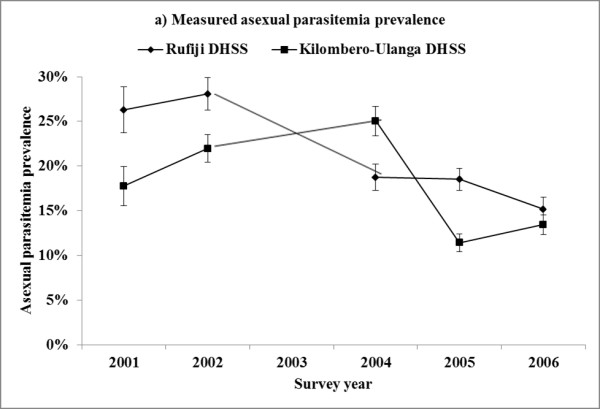
Measured (a) and modeled (b) asexual parasitaemia prevalence in Rufiji and Kilombero-Ulanga (KU) Demographic and Health Surveillance System (DHSS) sites, Tanzania, 2001, 2002, 2004, 2005, 2006 (N = 37,309).

Asexual parasitaemia prevalence varied from 11 to 28% (Table 1) and decreased from 23% to 14% (−9%-points) in Rufiji between 2001–2002 and 2004–2006, and from 18% to 14% (−4%-points) in Kilombero-Ulanga between 2001–2002 and 2004–2006, respectively. The change in intervention site minus the change in the comparison site was −5 percentage-points or −23%. Using a logistic regression model with terms for site, survey year and intervention (i.e. Rufiji in 2004–2006), age group, socioeconomic status, untreated bed net and ITN use, and rainfall in the six months before the interview date, the implementation of AS + SP was associated with a net decrease in asexual parasitaemia prevalence of 5 percentage-points or a relative decrease of 23% (Table 3, Figure 1). Gametocytaemia prevalence was low, ranging from <1% to 2%, and was not significantly associated with the implementation of AS + SP in Rufiji (p = 0.30) (Table 1, Table 4).

**Table 3:** Predictors of asexual parasitaemia in Rufiji and Kilombero-Ulanga (KU) Demographic and Health Surveillance System sites, Tanzania, 2001, 2002, 2004, 2005, 2006 (N = 37,309)

**Variable**	**Estimate**	**p-value**	**Adjusted odds ratio (95% confidence interval)**
**Year of survey**			
2001	Referent	Referent	Referent
2002	0.1457	0.0966	1.157 (0.974–1.374)
2004	0.0978	0.2319	1.103 (0.939–1.294)
2005	−0.3882	<0.0001	0.678 (0.480–0.793)
2006	−0.4058	<0.0001	0.666 (0.566–0.785)
**Demographic and health surveillance system site**			
KU	Referent	Referent	Referent
Rufiji	0.3092	<0.0001	1.362 (1.174–1.580)
**Implementation of artesunate-sulphadoxine-pyrimethamine**	−0.3366	<0.0001	0.714 (0.619–0.823)
**Age group**			
<1	0.8873	<0.0001	2.428 (2.059–2.864)
1- < 5 years	1.7099	<0.0001	5.529 (5.106–5.986)
5–15 years	1.5928	<0.0001	4.918 (4.580–5.280)
>15 years	Referent	Referent	Referent
**Bed net use previous night**			
No net use	Referent	Referent	Referent
Used untreated net	−0.2893	<0.0001	0.749 (0.689–0.814)
Uses insecticide treated net	−0.2590	<0.0001	0.772 (0.696–0.856)
**Wealth quintile**			
Poorest	0.5331	<0.0001	1.704 (1.520–1.911)
Less poor	0.4862	<0.0001	1.626 (1.453–1.820)
Middle	0.4816	<0.0001	1.619 (1.453–1.804)
More rich	0.3436	<0.0001	1.410 (1.266–1.571)
Least poor	Referent	Referent	Referent
**Rainfall**			
Rainfall in the 6 months before interview (per cm)	0.000272	0.6653	1.000 (0.999–1.002)

**Table 4:** Predictors of gametocytaemia in Rufiji and Kilombero-Ulanga (KU) Demographic and Health Surveillance System sites, Tanzania, 2001, 2002, 2004, 2005, 2006 (N = 37,309)

**Variable**	**Estimate**	**p-value**	**Adjusted odds ratio (95% confidence interval)**
**Year of survey**			
2001	Referent	Referent	Referent
2002	0.0364	0.8929	1.037 (0.610–1.763)
2004	−0.2546	0.2988	0.775 (0.480–1.253)
2005	−0.9695	<0.0001	0.379 (0.238–0.604)
2006	−1.2736	<0.0001	0.280 (0.165–0.476)
**Demographic and health surveillance system site**			
KU	Referent	Referent	Referent
Rufiji	−0.1957	0.3763	0.822 (0.533–1.268)
**Implementation of artesunate-sulphadoxine-pyrimethamine**	−0.2334	0.2961	0.792 (0.511–1.227)
**Age group**			
<1	1.7143	<0.0001	5.553 (3.376–9.133)
1- < 5 years	2.0611	<0.0001	7.854 (5.886–10.481)
5–15 years	1.0336	<0.0001	2.811 (2.061–3.834)
>15 years	Referent	Referent	Referent
**Bed net use previous night**			
No net use	Referent	Referent	Referent
Used untreated net	−0.2650	0.0732	0.767 (0.574–1.025)
Uses insecticide treated net	−0.2774	0.1478	0.758 (0.520–1.103)
**Wealth quintile**			
Poorest	0.6146	0.0013	1.849 (1.270–2.691)
Less poor	0.3059	0.1289	1.358 (0.915–2.015)
Middle	0.5591	0.0020	1.749 (1.227–2.494)
More rich	0.3651	0.0434	1.441 (1.011–2.053)
Least poor	Referent	Referent	Referent
**Rainfall**			
Rainfall in the 6 months before interview (per cm)	−0.00075	0.6850	0.999 (0.996–1.003)

## Discussion

Artemisinin-based combination therapy has been adopted as first-line anti-malarials for case management by most malaria-endemic countries. The change to ACT in most cases was prompted by the development of resistance to conventional anti-malarial monotherapies creating the need to implement a clinically efficacious drug and supported by the potential for reduced transmission and delayed development or spread of anti-malarial drug resistance. However, little is still known about the impact of introducing ACT on malaria endemicity as measured by asexual parasitaemia prevalence, especially in settings of high malaria transmission. The results of this large observational study document that implementation of AS + SP was associated with a modest decrease in the population asexual parasitaemia prevalence.

This evaluation design with a contemporaneous comparison group, allows adjustment for potential confounders, such as untreated net and ITN use, rainfall, and other factors that are associated with parasitaemia, such as age and socioeconomic status. Prior studies have demonstrated individual level clinical efficacy of ACT and some studies have suggested reduced malaria burden with ACT implementation [10-14], but to date there has been little evidence for the independent contribution of ACT-based case management over and above vector control interventions. This study is the first to show an independent association between ACT implementation and malaria endemicity after controlling for other malaria interventions such as ITN use, environmental factors such as rainfall, and other secular trends.

These findings suggest it is plausible that the introduction of AS + SP contributed to the reduction in asexual parasitaemia prevalence is based on an ecological association between the introduction of AS + SP and decreased asexual parasitaemia prevalence. After implementation of AS + SP, 20 to 87 AS + SP treatments per 100 persons per year were delivered through fixed health facilities. Although this number varied substantially during the study time period and is based on individual recall and an underlying assumption that AS + SP use was constant throughout the year, it still represents substantial drug pressure on the population and could plausibly account for the decrease in parasitaemia prevalence. Indeed, the introduction of SP monotherapy at fixed health facilities in these sites between 2001 and 2002 exerted enough drug pressure to select for genotypes associated with clinical drug resistance [40]. A more detailed analysis of dispensing patterns in Rufiji, Kilombero and Ulanga Districts documenting over 271,953 clinician-patient encounters at health facilities supports the widespread use of AS + SP in our intervention site throughout the year [41]. In addition, AS + SP is likely more efficacious at clearing parasitaemia than SP alone based on *in vivo* results collected from these sites over the same time period as well as published reports from elsewhere in Tanzania [42-46].

A modest absolute reduction in asexual parasitaemia prevalence of 5 percentage-points or a relative decrease of 23% occurred following the introduction of ACT in Rufiji. Since asexual parasitaemia prevalence was observed in all ages and the majority of parasitaemic persons were asymptomatic adults, this finding reflects the effect of ACT on asymptomatic carriage of parasites mostly in adults. The relationship between asexual parasitaemia prevalence and other disease burden estimates, such as uncomplicated or severe malaria incidence and mortality, is not addressed in this study. Previous mathematical models suggest an appreciable reduction in uncomplicated malaria incidence with the introduction of ACT, thus although the true magnitude of the reduction in malaria morbidity and mortality that might have been associated with this intervention cannot be quantified, the modest reduction in malaria endemicity noted in this study does not undermine the large public health impact of ACT.

Although a modest reduction in malaria endemicity was demonstrated, several mechanisms by which the introduction of ACT might reduce population level asexual parasitaemia prevalence are possible. The effect on population level parasitaemia prevalence might be related to the ACT’s superior efficacy at the individual level alone. In addition, the gametocytocidal effects of artemisinins could contribute, as has been suggested by individual-level studies [3-8,47,48]. It was not possible to support this claim with the population-level gametocytaemia prevalence reported here, as the data were limited by sample size and the low prevalence (~1%) of gametocytaemia among asymptomatic community members. Moreover, in this area of high transmission intensity and frequent AS + SP use, the decrease in population level parasitaemia might be due to the post-treatment prophylactic effect of SP. However, since both sites used SP, it is unlikely that the prophylactic effect would contribute to reduced parasitaemia prevalence differentially in Rufiji versus Kilombero-Ulanga. Lastly, mathematical models suggest that the introduction of even highly gametocytocidal ACT for case management of symptomatic patients with uncomplicated malaria will have only a modest impact on malaria endemicity, because of the presence of a large reservoir of asymptomatic patients who can sustain high levels of transmission and the rapid re-infection of patients who had been recently treated with ACT. In addition, the gametocytocidal properties of artemisinin drugs appear limited to immature sexual stages and mature gametocytes present at the time of treatment may persist and perpetuate transmission even after successfully completing an ACT regimen [49]. In sum, although one or more of several possible mechanisms may contribute, the modest reduction in parasitaemia prevalence found in this study is in line with reductions predicted by mathematical models of case management using effective artemisinins with rapid schizonticidal activity, gametocytocidal properties and post-treatment prophylactic effect [9].

### Limitations

This study had a number of limitations. The observational nature of this study leaves the analysis unable to control or even measure all potential confounders. On the other hand, the presence of a contemporaneous comparison area did allow consideration of known environmental factors such as rainfall and the use of malaria control interventions such as ITNs. Although randomization and blinding were not practical, the sites are ecologically similar and were observed over a period of several years. Prior research in both sites suggests similar malaria transmission intensity, although differences in malaria intervention coverage were present as noted. In addition, the quantification of SP monotherapy and AS + SP drug pressure may be imprecise as it relied on individual recall and the assumption that anti-malarial access, uptake and use were constant throughout the year while the survey was conducted following peak malaria transmission season each year. In particular, AS + SP use was likely underestimated for Rufiji in 2006 since the site experienced a localized stockout of AS at the time of the survey, following delayed implementation of the National Malaria Control Programme’s plans to transition to artemether-lumefantrine as a first-line anti-malarial. However, even rough estimates suggest substantial AS + SP drug pressure on the population and suggest it is plausible that the implementation of AS + SP contributed to the reduction in parasitaemia prevalence. Lastly, we relied solely on light microscopy to measure gametocytaemia, which most likely yields lower prevalence than could be detected via newer means such as molecular assays [50]. This robust data set with observations over five years using a pre-post evaluation design with a non-randomized contemporaneous comparison design may offer one of the best opportunities to observe the impact of ACT policy in a real-world context. Given the increased use of ACT worldwide it is unlikely that there will be another such opportunity to compare ACT and non-ACT case management policies on such a scale.

## Conclusions

A large observational study was used to assess the impact of introducing ACT on population-level malaria parasitaemia prevalence. This study quantifies the contribution of ACT-based case management policies in reducing malaria endemicity. Many countries in sub-Saharan Africa have implemented ACT, based on clinical efficacy alone and the promise of decreased transmission and reduced risk of developing resistance. This evaluation sought to measure the impact of such a policy change on malaria endemicity in a typical health system facing high levels of malaria transmission. Findings reported here provide the strongest evidence to date that case management with ACT can have a modest independent impact on malaria endemicity, but is unlikely to markedly reduce malaria transmission in high transmission settings such as most of sub-Saharan Africa.

## Competing interests

The findings and conclusions presented in this manuscript are those of the authors and do not necessarily reflect the official position of the US Centers for Disease Control and Prevention. The authors declare that they have no competing interests.

## Authors’ contributions

RAK contributed to the design of the study, supervised the field surveys, analysed and interpreted the data, and wrote the manuscript in consultation with the other authors. JS, BFE, JMR, JRG, AMwita, BG, MT, AMills and HM contributed to the analysis and interpretation of the data and to the drafting and editing of the manuscript. JDN, CG, EK, JRM, AMK, EES, MFS, and TL assisted in the design of the study, execution of the field surveys, interpretation of the data and editing of the manuscript. PBB, SA and SPK oversaw all aspects of the study, including design and execution of the field work, analysis and interpretation of the data and drafting of the manuscript. SPK drafted the protocol. All authors read and approved the final manuscript.
